# Recruiting for high reliability: attracting safety-minded applicants through language on company webpages

**DOI:** 10.1080/00049530.2023.2195007

**Published:** 2023-04-03

**Authors:** Cati S. Thomas, Laura S. Fruhen, Serena Wee

**Affiliations:** School of Psychological Science, University of Western Australia, Perth, Australia

**Keywords:** Gendered language, high reliability organisations, person-organisation fit, recruitment messages, safety attitudes

## Abstract

**Objective:**

Job candidates are attracted to companies where they see their values fit in based on clues from recruitment materials. Safety-critical companies may aim to attract safety-minded applicants, through signals indicating that the organisation prioritises safety. Research shows that language related to safety outcomes (versus other outcomes) in recruitment materials can inform the preferences of safety-minded applicants. Rooted in theorising that high reliability organisations (HROs) are highly safety-focused and have low masculinity values, this study investigates the extent to which the relationship between company attractiveness and safety-focused and femininity-focused language used to describe the company, is moderated by potential applicants’ safety attitudes.

**Method:**

In a within-subjects vignette study, participants (*N* = 197) rated the attractiveness of four fictitious companies, based on company webpages, and reported on their individual safety attitudes.

**Results:**

Participants with higher safety attitudes rated companies as more attractive when language used in company descriptions focused on safety (and not business). This effect was amplified when the company description also emphasised low masculinity (i.e., feminine) values.

**Conclusions:**

To attract applicants with higher safety attitudes, companies may benefit from using language that is focused on femininity, in addition to safety.

## KEY POINTS

**What is already known about this topic:**

(1) Language in recruitment materials can be used to convey informational signals about organisational values.
(2) Person-organisation fit (P-O fit) is the compatibility between people and companies when they share similar fundamental characteristics; P-O fit predicts applicant attraction.
(3) Using safety-focused language appears to be attractive for applicants who are more safety-minded.

**What this topic adds:**

(1) Further evidence that safety-focused language in company descriptions would be attractive to safety-minded applicants.
(2) Femininity-focused language is also attractive to safety minded applicants.
(3) Femininity- and safety-focused language work together in attracting safety-minded applicants.

Globally, more than 2.78 million people are estimated to die each year due to work-related injuries or diseases (International Labour Organization, 2020). In Australia, work-related fatalities have increased between 2018 and 2019 (Safe Work Australia, 2019). Safe operations result from the interplay between person and environmental factors, and can be shaped through strategic selection of individuals with particular attributes (Hobbs & Williamson, 2002; Vredenburgh, 2002). In this study, we focus on how companies could attract job applicants who are likely to prioritise safety. In attracting safety-minded applicants, workplaces can ensure they recruit safety conscious individuals (i.e., person factors), who in turn contribute to a workplace in which safety-focus is the norm (i.e., environment factors).

Previous research has shown that job advertisements with safety (vs. business) messaging are more attractive for individuals with higher safety attitudes (Fruhen et al., 2015). The study used phrases such as “*Managing our organisation safely”* and “*Guarantees safety for all our employees*” to construct job advertisements prioritising safety. In this paper, we extend the findings by Fruhen et al. (2015) by examining language used in company webpages that is femininity- versus masculinity-focused as an additional attribute relevant to attracting safety-minded applicants. Our consideration of femininity-focused language is informed by theorising on High Reliability Organisation (HROs). HROs — which include nuclear power plants, air traffic management and many airlines — strongly prioritise reliability and safety (Weick et al., 2008). These organisations operate in environments where errors can have catastrophic consequences: endangering lives, destroying equipment, and seriously harming the environment (Francisco et al., 2020; Weick et al., 2008). Yet, HROs operate safely in such environments. A key aspect of their capacity to achieve excellent safety outcomes, beyond having a strong priority for safety, has been attributed to the undoing of masculine gender norms.

Employees in HROs express low masculinity values by admitting physical limitations (Ely & Meyerson, 2010), asking for assistance (Weick & Sutcliffe, 2001), and correcting mistakes (Roth et al., 2006). Just as importantly, they do not engage in overly aggressive, competitive, or “macho” behaviours (Berdahl et al., 2018; Ely & Meyerson, 2010). Behaving in this way signals that the company values the avoidance of unnecessary risk-taking, emotional restraint and self-reliance (Ely & Meyerson, 2010), which has been shown to relate to positive safety outcomes (Austin & Probst, 2021). Thus, we argue that femininity-focused language can be used to signal that a company has low masculinity values. Given that HROs’ safety success is in part associated with low masculinity values, we aim to test if femininity-focused language on company webpages can further enhance the attraction of safety-minded applicants to safety-critical jobs.

In considering both femininity- and safety-focused language in company webpages, this study furthers our understanding of how specific word choices made in recruitment materials may support the attraction of safety-minded applicants. As previous research has shown, the *meaning* (or lexical definition) of the words chosen to describe a company affects how attractive that company is to safety-minded applicants (Fruhen et al., 2015). In the current study, we further show how the specific words chosen can, additionally, act as subtle cues that affect our judgements and evaluations of a person or organisation (Hancock & Dunham, 2001). For example, Hauser and Schwarz (2018) found that a target person was rated as less warm and competent when described as *utterly changed* rather than *totally changed*. This effect occurred even though participants perceived the adverbs to be synonymous (i.e., have the same lexical definition; Hauser & Schwarz, 2018). Research suggests socially learned meanings are associated with the chosen words that uniquely impact the impressions that are formed (Hauser & Schwarz, 2018; Hosman & Siltanen, 2006; Smith et al., 1998), including how attractive something is (Hosman, 2002).

To examine how word choice impacts organisational attraction, we test the extent to which words used in company descriptions – which may subtly cue a femininity (e.g., *supporting*) versus masculinity focus (e.g., *leading*), or cue a safety (e.g., *safely*) versus business focus (e.g., *competitively*)— increase the attractiveness of a company to safety-minded applicants. We incorporate impression formation theory into theoretical perspectives of how to attract and hire individuals with attitudes and mindsets that fit an organisation’s norms and values (Cable & Judge, 1996; Edwards & Cable, 2009). In what follows, we describe two theoretical perspectives (person-organisation fit [Kristof, 1996] and signalling theory [Rynes et al., 1991; Spence, 1974]), and elaborate on how each explains how word choices may induce attraction in applicants.

## Person-organisation fit, signalling theory and attraction to companies

Person-organisation fit (P-O fit) is the perceived compatibility between people and companies when they share similar fundamental characteristics (Kristof, 1996). P-O fit theory proposes applicants will respond positively to companies with values and norms that align with their own (Swider et al., 2015; Van Vianen et al., 2012), and applicant attraction is the expected outcome of a strong perceived P-O fit (Dineen et al., 2002). In fact, applicants tend to prefer companies whose values closely align with their own (Cable & Judge, 1996), and a meta-analysis of 232 studies has found that the strongest predictors of applicant attraction were perceived job and organisational fit (Uggerslev et al., 2012).

As described by Rynes et al. (1991) in their influential paper on signalling theory, company values can be signalled, through explicit and more subtle cues (see also Celani & Singh, 2011 for a multi-level extension). Theoretically, it should be those candidates with values, attitudes, and norms matching a prospective company’s that would be the most strongly attracted by that company’s signals. For example, research by Turban and Keon (1993) found that when information about performance-based pay was signalled, participants with a higher need for achievement were more interested in these companies. By focusing on the language used to signal values to applicants, we bring theorising and research insights from impression formation theory into the context of signalling theory (Hauser & Schwarz, 2018). Specifically, we suggest that the recruitment cue, signalled through different word choices, serves to form a view of a company that leads applicants to be more attracted when they perceive the organisational values to align with their own. For safety, explicitly signalling in the job advertisement that safety is prioritised attracts safety minded job applicants (Fruhen et al., 2015). This finding aligns with research on the role of language in impression formation (e.g., Hancock & Dunham, 2001; Hauser & Schwarz, 2018). Our contribution suggests it is not just the explicit, content cue of the words that matter, but that the connotations and impressions signalled by the chosen word also matters (i.e., *supporting* connotes—but does not explicitly indicate—safety values).

## Recruiting for safety

**Figure 1.**
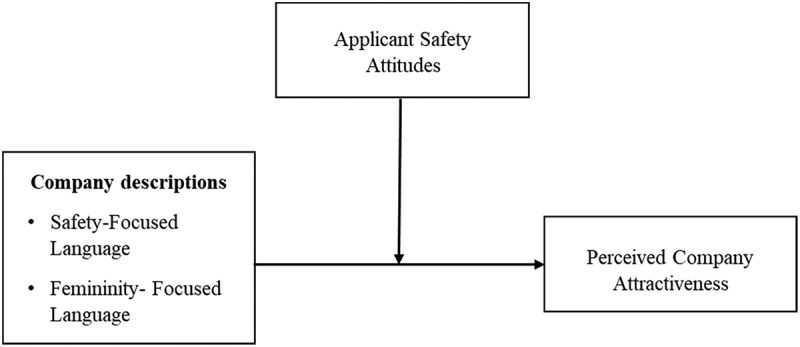
Summary of the proposed relationships among variables examined in the study.

Applying the principles of P-O fit (Kristof, 1996) and signalling theory (Rynes et al., 1991; Spence, 1974) to the attraction of safety-minded applicants, we focus on the attraction of applicants with higher (vs. lower) safety attitudes. Safety attitudes capture a person’s beliefs and emotions regarding safety policies, procedures, and practices (Neal & Griffin, 2006). Safety attitudes inform safety related behaviour and performance and relate to the accident history of individuals (Hofmann et al., 1995; Mearns et al., 1998). By drawing upon impression formation research (Hancock & Dunham, 2001; Hauser & Schwarz, 2018), we examine how language in company descriptions impact applicant attraction based on their safety attitudes. The relationships proposed in our hypotheses are summarised in Figure 1.

In particular, the use of safety-focused language rather than business-focused language is expected to convey company priorities concerning safety. Word choice has generally been identified as informing our view of others (Hauser & Schwarz, 2018). For example, the meaning (i.e., lexical definition) of some words inherently convey valence (e.g., *good*, *evil*). However, other words exist that are not inherently valanced, yet come to hold valanced meaning because they tend to be used within certain contexts that tend to be valanced (Sinclair & Sinclair, 1991). For example, Hauser and Schwarz (2018) suggest that someone *causing* work would be viewed more negatively than someone *producing* work, even though *causing* and *producing* are often considered synonymous. This is because *causing* work is often interpreted negatively as creating additional work that is burdensome in nature. In contrast, *producing* work is often interpreted positively as delivering on an opportunity. This effect is theorised to occur because the meaning of words is learned through social interaction, so that even when words do not have an inherent valence, we learn to interpret them in valanced ways (Hauser & Schwarz, 2018).

The differential impact of word choice has been shown in previous recruitment-related research: safety-focused language has been shown to signal the extent to which safety is a priority for a company (Fruhen et al., 2015), and gendered language to signal gender stereotypes that contribute to gender inequality in workplaces (Gaucher et al., 2011). According to P-O fit theory (Swider et al., 2015; Van Vianen et al., 2012), potential applicants with high safety attitudes, keen to seek out workplaces that match their personal values, will be more attracted to workplaces that signal similar priorities around safety. With only one study to date testing this effect, we propose to replicate the findings by Fruhen et al. (2015) and hypothesise:

H1: Companies that convey a safety focus in company webpages will be more attractive to applicants with high safety attitudes compared to companies with a business focus.

Second, extending the theorising around the role of language in impression formation (Hauser & Schwarz, 2018) and specifically considering theorising about the role of femininity for safety outcomes (Austin & Probst, 2021; Ely & Meyerson, 2010), companies may also attract applicants with more positive safety attitudes by signalling femininity in addition to strong safety values. While not directly addressing safety, feminine language may convey the extent to which a company values safety beneficial practices such as speaking up, help-seeking, or avoiding risk taking (Austin & Probst, 2021). Specifically, femininity- focused language, is proposed to *additionally* signal values that should be more attractive to applicants with high safety attitudes, given the described connection of high safety and low masculinity values in the HRO literature (Ely & Meyerson, 2010). We propose that the addition of femininity (vs. masculinity) signals in language will make safety-focused companies even more attractive to safety-minded applicants. We therefore hypothesise:

H2: Companies that convey a safety focus and femininity focus will be more attractive to individuals with high safety attitudes, compared to companies that convey a safety focus and masculinity focus.

Next, we also consider the extent to which feminine language, compared to masculine language, will be effective in attracting safety-minded applicants when safety values are not directly signalled through safety-focused language. As femininity-focused language can signal that a company values safety beneficial practices (Austin & Probst, 2021), we propose that even in the absence of direct safety messaging, this effect will occur, albeit in a less pronounced form compared to when safety-focused language is also used. Accordingly, we predict there will be an effect of femininity-focused language on attracting safety-minded applicants when comparing companies signalling a business focus alongside a femininity focus, with companies signalling a business focus alongside a masculinity focus. But we expect this effect to be smaller. We propose the effect of undoing gender is somewhat dependent on a safety focus given the central function of safety focus in HRO theorising. We hypothesise:

H3: Companies that convey a business focus and femininity focus will be more attractive to individuals with high safety attitudes, compared to companies that convey a business focus and masculinity focus.

## Method

### Participants and procedure

The sample consisted of 197 participants (of which 128 [65.0%] were female). Participants were, on average 32.05 years (*SD* = 9.99, range: 19–69), with the majority (67.5%) employed in full or part-time work, 15.2% were students, 9.1% were casually employed, and 8.1% were unemployed.

This study was approved by the University of Western Australia’s Human Research Ethics Committee RA/4/20/6147. Participants were recruited through advertisement of the study on LinkedIn using snowball methods. Participants had the option to enter a prize draw to win one of three $50 gift cards for their time.

We excluded participants if they indicated their data should not be used (*n* = 5), if they did not identify as either male or female or preferred not to provide a response about their gender (*n* = 9), or if they responded implausibly fast (*n* = 32), based on the average English word reading speed reported by Brysbaert’s (2019) meta-analysis. This criterion was adopted as rushing could indicate careless responding (Huang et al., 2012).

### Materials

#### Vignettes

**Figure 2.**
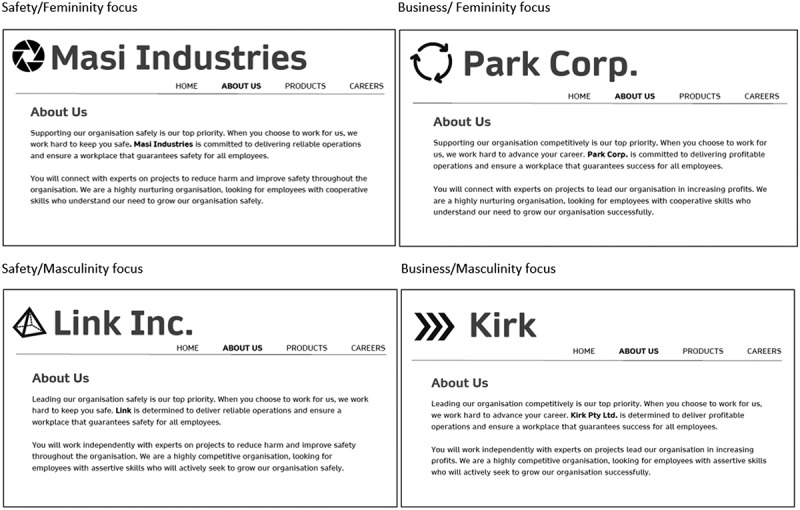
Descriptions on company webpages varying safety-focused (vs. business-focused) language, and femininity-focused (vs. masculinity-focused) language.

Participants were randomly presented with vignettes of four “About Us” sections of fictitious company webpages in a within-subjects-design. The vignettes showed webpage content, rather than job advertisements, as this medium is the main information source for job seekers forming impressions about a company’s culture (Acikgoz, 2019; De Goede et al., 2011). The four vignettes were modelled on the descriptions used in Fruhen et al.’s study (2015) and constructed almost identically with company names and a logo (See Figure 2).

The safety versus business and femininity versus masculinity foci conveyed in the company webpages were manipulated via the language used. Five safety and five business-related words and phrases were obtained from Fruhen et al.’s study (2015) to convey a safety and a business focus respectively. Six words and phrases signalling a masculinity versus a femininity focus were obtained from Gaucher et al. (2011) to create the femininity-focused language manipulation (see Table 1 for all varied words or statements).

**Table 1.** List of words and phrases used to convey the four language foci.

**Safety-focused language manipulation**	
Business-focused	Safety-focused
Competitively Advance your career Profitable Success* Lead our organisation in increasing profits	Safely* Keep you safe Reliable Safety Reduce harm and improve safety throughout the organisation
**Femininity-focused language manipulation**	
**Masculinity-focused**	**Femininity-focused**
Leading	Supporting
Determined	Committed
Work independently	Connect
Competitive	Cooperative
Assertive	Nurturing
Actively	Understand

*Indicates word was used twice.

#### Pilot study for vignette validation

A pilot study was conducted to validate the extent to which vignettes signalled safety versus business foci, and femininity versus masculinity foci. A sample of 75 participants were recruited from MTurk, of which 42 (56%) identified as male. Participants were aged from 22 to 72 years (*M* = 40.60, *SD* = 12.38), with the majority (69.3%) employed full-time, 16% were employed part-time or on a casual basis, and 14.6% were unemployed or full-time students.

Pilot study participants rated their perception of each vignette to demonstrate a focus: safety, business, masculinity, and femininity. This was rated on a 5-point Likert scale (1=Strongly disagree to 5=Strongly agree) with higher scores indicating a stronger safety, business, masculine or feminine focus. Results indicated that the safety versus business language manipulation was effective as there was a significant main effect of safety vs business language manipulation on safety-focus ratings of each company, *F*(1, 74) = 10.15, *p* = .002. Specifically, company webpages with high levels of safety language were perceived to be more safety-focused than webpages with business-focused language. Similarly, company webpages with masculinity-focused language were perceived to be more masculine-focused than those with femininity-focused language, *F*(1, 74) = 29.261, *p* < .01. Accordingly, participants were able to distinguish between the foci of the vignettes on both categories that were manipulated. Further results regarding the vignettes’ capacity to convey organisational values are reported in the Appendix. The results of the pilot suggest that the variation in language clearly indicated varying foci on safety versus business and masculinity versus femininity.

#### Survey measures

##### Organisational attractiveness

Three items from Highhouse et al.’s (2003) measure of organisation attractiveness were used in this study (Cronbach’s α = .87). Items such as *This company is attractive to me as a place of employment* were selected based on their relevance to the research question, and participants responded to each item using a 5-point Likert scale (1=Strongly disagree to 5=Strongly agree) and a mean score was computed. This measure possesses good construct validity as all items were reported to load onto a single factor (with loadings of .60 and above; Highhouse et al., 2003).

##### Safety attitudes

Henning et al.’s (2009) 6-item scale was used to assess participants’ attitudes towards safety by responding to the prompt “*In general I believe that in an organisation … ”*. Items such as *Safety should have a high priority* are answered on a 5-point Likert scale (1=Strongly disagree to 5=Strongly agree) with higher scores indicating a stronger attitude towards safety. Cronbach’s alpha was .86 and a mean score was computed. These items have been shown to load on a single factor (with loadings of .61 and above) in an exploratory factor analysis, suggesting that they have good construct validity and represent general safety attitudes (Henning et al., 2009).

#### Analytic approach

The descriptive statistics (means and standard deviations) reported in the results section assessed how participants rated the attractiveness of the four company webpages independent of their safety attitudes. The hypotheses were tested by conducting multilevel analyses in R (R Core Team, 2020) using the multilevel package (Bliese, 2016), to account for the nested nature of the data. Organisation attractiveness was the dependent variable. The within-person predictors were the experimentally manipulated language used to describe the company webpages, and the between-person predictors were individual-level mean-centred safety attitudes. Random intercept models were used to account for the within-person data structure (which accounted for 27.18% of the variance in company attractiveness). To facilitate the interpretation of the interaction effect, we followed-up with a simple slopes analysis (Preacher et al., 2006).

## Results

Independent of their safety attitudes, participants found the company that used safety-focused language together with femininity-focused language the most attractive (*M* = 3.57, *SD* = 0.86). This was followed by the safety- and masculinity-focused company (*M* = 3.35, *SD* = 0.90), the business- and femininity-focused company (*M* = 3.28, *SD=*0.94), and the business focus and masculinity-focused company (*M* = 3.01, *SD=*1.03).

Hypothesis 1 states that company webpages conveying a safety focus versus a business focus will be more attractive for individuals who have higher safety attitudes. The results from the multilevel analyses shown in Table 2 indicate a significant positive effect of safety attitudes on organisational attractiveness, that was qualified by a significant interaction with language focus. The simple slopes analyses indicate that for participants higher on safety attitudes (i.e., +1SD above the mean), safety-focused companies were more attractive than business-focused companies (γ = 0.424, *SE* = 0.079, *p* < .001), but for participants lower on safety attitudes (i.e., −1SD from the mean), safety-focused companies were as attractive as business-focused companies (γ = 0.098, *SE* = 0.079, *p* = .213).

**Table 2.** Fixed effects estimates for multilevel model testing the safety-focused language × safety attitude interaction on organisational attractiveness.

Fixed Effect	Estimate	*SE*	*P*
Intercept	3.20	0.05	< .001
Safety-focused Company	0.26	0.06	< .001
Safety Attitude	0.19	0.08	.015
Safety-focused Company × Safety Attitude	0.24	0.08	.004

*N* = 197.

**Figure 3.**
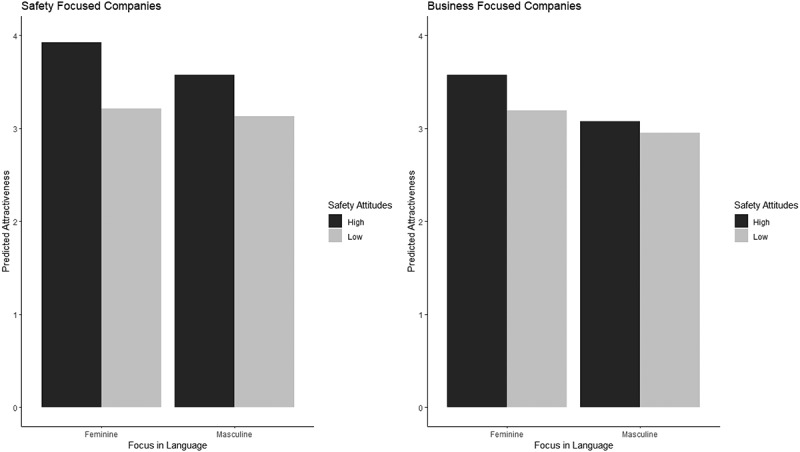
Cross-level interaction effect between femininity-focused language and safety attitudes on organisation attractiveness in safety-focused companies (Hypothesis 2) and business-focused companies (Hypothesis 3).

Hypothesis 2 states that company webpages that convey both a safety focus and femininity focus, will be more attractive to individuals with higher safety attitudes, compared to those companies that convey a safety focus and a masculinity focus. The results of the multilevel regression analyses reported in Table 3 demonstrate that, for safety-focused companies, participants with higher safety attitudes rated these companies as more attractive than participants with lower safety attitudes. Follow-up statistical tests indicate that for participants higher on safety attitudes, safety-focused companies that also had a feminine focus were more attractive than safety-focused companies that had a masculine focus (γ = 0.352, *SE* = 0.090, *p* < .001). For participants lower on safety attitudes, safety-focused companies that had a feminine focus were as attractive as safety-focused companies that had a masculine focus (γ = 0.078, *SE* = 0.090, *p* = .388). These results are presented in Figure 3 and provide support for Hypothesis 2.

**Table 3.** Fixed effects estimates for multilevel models testing the femininity-focused language × safety attitude interaction on organisational attractiveness.

Fixed Effect	Safety-focused Language			Business-focused Language		
	Estimate	*SE*	*p*	Estimate	*SE*	*p*
Intercept	3.35	0.06	< .001	3.01	0.07	< .001
Femininity-focused Language	0.21	0.06	0.001	0.37	0.07	< .001
Safety Attitude	0.33	0.09	< .001	0.09	0.10	.36
Femininity -focused Language × Safety Attitude	0.20	0.10	.036	0.18	0.10	.071

*N* = 197.

Hypothesis 3 states that companies that convey a business focus and femininity focus on their webpage, will be more attractive to individuals with higher safety attitudes, compared to those companies that convey a business focus and a masculinity focus on their webpage. The results presented below in Table 3 show that there was a significant effect of femininity-focused language on company attractiveness, but no significant effect of safety attitudes on attractiveness. There was also no significant interaction effect, and hence Hypothesis 3 was not supported. These results are also presented in Figure 3.

## Discussion

This study investigated how language on company webpages can convey two key aspects of HRO culture: a strong focus on safety and low masculinity norms (via a focus on femininity; Austin & Probst, 2021; Ely & Meyerson, 2010). Rooted in impression formation theory (Hauser & Schwarz, 2018) this study aimed to identify how language may influence applicants’ attraction to companies depending on their own safety attitudes. Results showed that applicants with higher safety attitudes were more attracted to companies that conveyed a safety focus on their company webpages than they were to companies that conveyed a business focus. This finding replicates Fruhen et al.’s (2015) results and further demonstrates how differing levels of safety focus in language can convey company priorities and attract potential applicants based on their safety attitudes. Overall, this finding is aligned with past research on P-O fit and attraction (Cable & Judge, 1996; Uggerslev et al., 2012), suggesting that in this study, people with higher safety attitudes saw greater P-O fit with companies described with a high safety focus.

Our results indicated that when applicants with higher safety attitudes rated the attractiveness of companies with a safety focus, they were more attracted to companies that also conveyed a femininity focus than to companies that also conveyed a masculinity focus. This result shows that organisations can amplify signalling a safety priority to job applicants by using language that conveys both safety and femininity. This finding aligns with theorising around HROs, for which a focus on safety as well as a preference for non-masculine role norms have been identified as central to their excellent safety track record (Austin & Probst, 2021; Ely & Meyerson, 2010). Our results further showed that femininity-focused language on company webpages only impacts the attraction of applicants with higher safety attitudes when combined with a focus on safety. We had hypothesised companies that convey a business focus and a femininity focus, will be more attractive to individuals with higher safety attitudes, compared to those companies that signal a business focus and a masculinity focus. The results showed that without a safety focus also being signalled by companies, femininity-focused language is not – by itself – sufficient to attract applicants with higher safety attitudes.

Taken together, our findings show that language in company webpages focused on safety, not business, can attract applicants with higher safety attitudes. This effect of a safety focus in language can be enhanced when espousing a focus on femininity, rather than masculinity. Overall, the findings illustrate the capacity of language to inform applicant attraction in relation to specific priorities (Braddy et al., 2006; Fruhen et al., 2015), which aligns with theorising about P-O fit (Kristof, 1996). With regards to theorising around HROs, the findings reinforce a focus on safety as a central pillar for HROs and at the same time illustrate that conveying low masculinity norms is an additional pillar (in line with theorising by Austin & Probst, 2021; Ely & Meyerson, 2010).

### Limitations and future research

Certain limitations should be considered regarding this study. First, the language manipulation in the companies’ webpages used Gaucher’s et al (2011) list of words and phrases. Although we verified that the webpages were perceived as intended, the companies’ webpages were not rated for authenticity and representativeness of an organisation’s actual webpage. This lack of realism may have impacted on attractiveness (De Goede et al., 2011; Überschaer et al., 2016). Second, because the vignettes were designed to be applicable to participants from a variety of educational and employment backgrounds, we did not provide additional information such as specifics about the industry or job role. In such situations, applicants are likely to be guided by their own industry expectations and stereotypes (De Goede et al., 2011) which may in turn influence their attraction.

Finally, our results may also lend themselves to future research into attraction of employees based on their masculinity values. In Australia, where recent inquiries have pointed to hyper-masculine cultures in some industries (Western Australian Community Development and Justice Standing Committee, 2022), using gendered language to attract individuals may facilitate organisational culture change. According to Hofstede’s dimensional model of national culture (Hofstede & Hofstede, 2001; Hofstede et al., 2005), Australia has a pronounced masculinity culture driven by competition, achievement, and success, and where men are expected to be tough and assertive. These cultural attributes suggest that the specific nature of the Australian context may be relevant to interpreting our findings. Future research should consider how wider societies’ masculine and individualistic cultural values may influence the extent to which candidates’ job preferences are swayed by femininity and safety focused language. Our study, being set in just one cultural context can be extended in future research by testing the identified relationships in other cultural contexts. Future research may also consider how changes in workplace culture may be supportive of wider cultural change in society more broadly around the issues focused on in this study.

### Practical implications

This study’s findings have practical implications for recruiting in safety-critical contexts. Companies that value safety may boost their attraction of safety-minded applicants by signalling both a safety and femininity focus. Doing so can assist with attracting applicants by matching their values and priorities around safety. Companies should be mindful of the extent to which the espoused values on their webpages match with their actual focus in their organisation. Value incongruence between an organisation and employees impacts satisfaction, commitment, and turnover intentions (Ostroff et al., 2005). That is, companies may be able to attract more safety-minded applicants by espousing a focus on safety and femininity. However, if these values are not actually reflected in the organisation’s practices, it is unlikely these employees will stay with the organisation for long or that attracting them in the first place will work as a strategy to change the workplaces’ priorities. A sole focus on attracting more safety-minded applicants to facilitate culture change is doomed to fail and will likely backfire by reducing commitment and having other unintended side effects (Howell et al., 2012). While clearly not easily achieved, culture change within workplaces around gender and safety issues is highly topical in the Australian context, where sexual harassment has been identified as an issue at work (Western Australian Community Development and Justice Standing Committee, 2022) and new health and safety laws and codes of practice have reaffirmed a focus on safety at work.

## Conclusion

This study enhances the understanding of how applicant attraction is influenced by language focused on safety and femininity. The results demonstrate the capacity of language to convey organisational priority and to attract applicants with higher safety attitudes to companies that prioritise safety. Companies such as HROs may further boost their likelihood of attracting applicants who have higher safety attitudes by intentionally selecting language in their recruitment materials that conveys a safety focus and femininity focus, rather than a focus on business and masculinity.

## Data sharing request

Due to the nature of this research, participants of this study did not agree for their data to be shared publicly, so supporting data is not available.

## Appendix

Beyond the perceptions of the language foci in company descriptions, we also tested to what extent the language manipulation was seen to convey company values in the pilot study. Participants rated the companies’ safety climate (Neal et al., 2000) by responding to the prompt, “*In this organisation* *…* ”. Three items such as “*Safety is given a* *high priority by management*” were answered on a 5-point Likert scale (1=Strongly disagree to 5=Strongly agree) with higher scores indicating the organisation is portraying a stronger safety climate. Cronbach’s alpha was .82. Participants were also asked about the masculinity norms conveyed in the company descriptions using a modified and shortened version of Oransky and Fisher’s (2009) masculinity norms scale by responding to the prompt “*In this organisation* *…* ”. The four selected items such as “*A guy must always appear confident even if he isn’t*” are applicable to the workplace. Items were answered on a 5-point Likert scale (1=Strongly disagree to 5=Strongly agree) with higher scores indicating the organisation is portraying stronger masculine norms. Cronbach’s alpha was .92 and a mean score was computed.

Results showed that company webpages with high levels of safety language were rated higher in their safety climate (*M* = 4.62, *SD*=.55) than company webpages with business language (*M* = 2.51, *SD*=.95); *F*(1, 74) = 296.57, *p* < .001. Also, company webpages with masculine language were rated lower in their safety climate (*M* = 3.49, *SD* = 1.35) than webpages with feminine language (*M* = 3.54, *SD* = 1.27); *F*(1, 74) = 8.70, *p* = .004. Further, company webpages with masculine language were rated higher in their masculinity norms (*M* = 3.10, *SD* = 1.05) than company webpages with feminine language (*M* = 2.51, *SD*=.93); *F*(1, 74) = 48.33, *p* < .001. And company webpages with safety-focused language were rated lower in their masculinity norms (*M* = 2.60, *SD*=.93) than webpages with business-focused language (*M* = 3.02, *SD* = 1.09); *F*(1, 74) = 18.303, *p* < .001. The results show that these foci were seen as clues to company values.
